# Spindle assembly checkpoint inactivation fails to suppress neuroblast tumour formation in *aurA* mutant *Drosophila*

**DOI:** 10.1038/ncomms9879

**Published:** 2015-11-16

**Authors:** Renaud Caous, Aude Pascal, Pierre Romé, Laurent Richard-Parpaillon, Roger Karess, Régis Giet

**Affiliations:** 1 Institut de Génétique et Développement de Rennes-Université de Rennes I-CNRS- UMR 6290, 2 avenue du Pr Léon Bernard, 35043 Rennes, France; 2 Institut Jacques Monod-Université Paris Diderot-Paris 7, 15 rue Hélène Brion, 75205 Paris, France

## Abstract

Tissue homeostasis requires accurate control of cell proliferation, differentiation and chromosome segregation. *Drosophila sas-4* and *aurA* mutants present brain tumours with extra neuroblasts (NBs), defective mitotic spindle assembly and delayed mitosis due to activation of the spindle assembly checkpoint (SAC). Here we inactivate the SAC in *aurA* and *sas-4* mutants to determine whether the generation of aneuploidy compromises NB proliferation. Inactivation of the SAC in the *sas-4* mutant impairs NB proliferation and disrupts euploidy. By contrast, disrupting the SAC in the *aurA* mutant does not prevent NB amplification, tumour formation or chromosome segregation. The monitoring of Mad2 and cyclin B dynamics in live *aurA* NBs reveals that SAC satisfaction is not coupled to cyclin B degradation. Thus, the NBs of *aurA* mutants present delayed mitosis, with accurate chromosome segregation occurring in a SAC-independent manner. We report here the existence of an Aurora A-dependent mechanism promoting efficient, timed cyclin B degradation.

## Abstract (web summary)

*Drosophila aurA* mutants develop brain tumours which are associated with defective mitotic spindle assembly. Caous *et al*. show that these mutants are surprisingly insensitive to tumour-suppressive spindle assembly checkpoint inactivation, due to a checkpoint-independent delay in cyclin B degradation.

## Graphical abstract

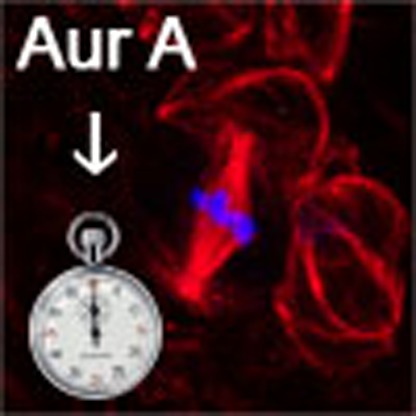

The mitotic spindle is a microtubule-based structure involved in the segregation of the two sets of duplicated chromosomes in the two daughter cells. In polarized fly neural stem cells (neuroblasts (NBs)), it is also involved in the differential segregation of cell fate determinants anchored to the apical and basal cell cortex. In this type of cell division, the two daughter cells have different fates: one retains NB identity and continues to proliferate, whereas the other differentiates. Defects in spindle orientation lead to the abnormal delivery of cell fate determinants. Consequently, both daughter cells may become NBs, leading to tumour formation^1^,^2^,^3^. This is the case for *sas-4* mutant flies, which have no centrosomes and display severe spindle orientation defects^4^,^5^. Spindle orientation and assembly are therefore tightly linked in the maintenance of tissue homeostasis and euploidy in the fly central nervous system (CNS). An understanding of the mechanisms involved in the defective asymmetric division of NBs is therefore relevant to cancer stem cell research^1^.

Aurora A kinase has been implicated in the formation of human cancers and is considered to be an oncogene^6^. Paradoxically, *Drosophila* Aurora A can also act as a tumour suppressor during the asymmetric division of NBs^7^,^8^,^9^. Mutations of *Drosophila aurA* result in the production of abnormal mitotic spindles with defective astral microtubule attachments to the apical cortex, leading to spindle misorientation, abnormal daughter cell fate acquisition and expansion of the NB population^7^,^8^,^9^,^10^,^11^. The transplantation of mutant *aurA* brains into the abdomens of host flies leads to tumour formation^5^. The spindle defects of *aurA* mutant NBs also lead to delays in mitotic progression. Wild-type NBs exit mitosis ∼6–7 min after nuclear envelope breakdown (NEBD), whereas *aurA* hypomorphic mutant neuroblasts do not exit mitosis until ∼20 min after NEBD. This delay is presumably mediated by prolonged spindle assembly checkpoint (SAC) activation in response to incorrectly attached kinetochores^4^,^7^,^12^.

In human cancer cells, weakening of the SAC compromises chromosome segregation and leads to massive aneuploidy and cell death^13^. We show here that the absence of the SAC in *sas-4* mutants strongly impairs chromosome segregation, euploidy and the ability of neural tissues to proliferate and to induce tumours. However, chromosome segregation and cell ploidy are unaffected by the absence of the SAC in *aurA* mutants, and the mutant tissue can still induce tumours following their injection into host flies. Our results suggest that impaired cyclin B degradation compensates for the defect in chromosome segregation in *aurA* neural tissues, in the absence of the SAC.

## Results

### SAC inactivation does not impair *aurA* NB amplification

We investigated the effects on fly viability and brain development of an absence of the SAC in *aurA*^*8839*^ null flies (hereafter referred as *aurA*). Mad2 is a key component of the SAC apparatus and appears to have no other role in *Drosophila*^14^.[Fig f1]

**Figure 1 f1:**
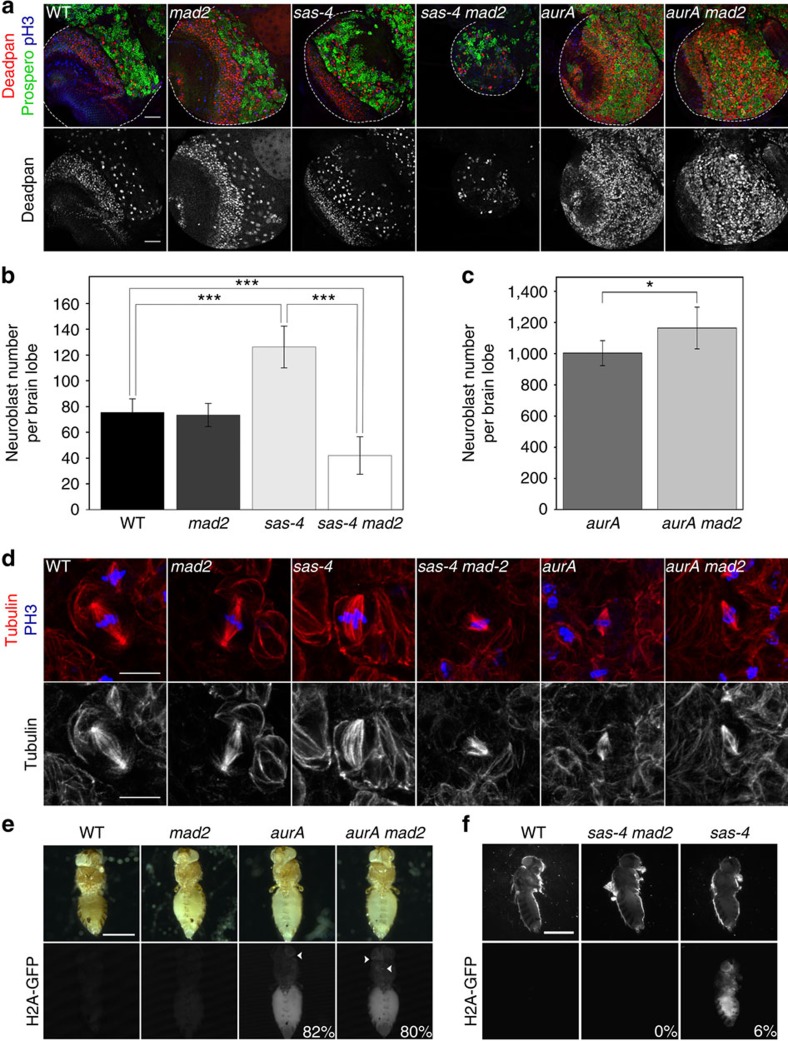
SAC deletion prevents the amplification of *sas-4* mutant NBs but not of *aurA* mutant NBs. (**a**) Examination of WT, *sas-4*, *aurA*, *mad2*, *sas-4 mad2* and *aurA mad2* mutant brain development. WT and *mad2* mutant brains grew normally, whereas *sas-4* and *aurA* mutant brains contained large numbers of NBs. SAC deletion impaired brain development in combination with *sas-4*, but not with *aurA*. The pan-NBs were counted by staining of the central brain region with an anti-deadpan antibody (red and lower monochrome panels). Staining for the neuronal marker prospero is shown in green. Staining for phosphorylated histone H3 (Ser10) is shown in blue. Scale bar, 50 μm. (**b**) Quantification of pan-NBs (±s.d.) in WT, *sas-4*, *mad2* and *sas-4 mad2* brain lobes. WT and *mad2* brain lobes had 75.4±10.5 NBs per lobe (*n*=14) and 73.2±9.0 NBs per lobe (*n*=22), respectively. sas-4 lobes contained a larger number of NBs per lobe (126.2±16.2 NBs; *n*=18). The disruption of Mad2 in a *sas-4* background decreased the growth rate and number of NBs (42.0±14.5, *n=*14). ***P<10^−10^ (Wilcoxon test). (**c**) Quantification of pan-NBs (±s.d.) in *aurA* and *aurA mad2* brain lobes. The number of NBs in *aurA* brain lobes (1003.6±79.7, *n*=12) was high, even in the absence of Mad2 (1164.2±134.3, *n*=11). *P<3 × 10^−3^ (Wilcoxon test). (**d**) SAC deletion compromises spindle morphology in *sas-4* but not *aurA* mutant NBs. Metaphase NBs from the indicated genotypes were stained for phosphorylated histone H3 (Ser10) (blue), tubulin (red and lower panels in monochrome). Many spindles in *sas-4* brains were bipolar, but *sas-4 mad2* mutant spindles were abnormal in shape. The *aurA* and *aurA mad2* mutants had short bipolar spindles. Scale bar, 10 μm. (**e**) The ability of *aurA* mutant brains to develop tumours is not compromised by SAC deletion. WT, *aurA*, *mad2* and *aurA mad2* brains were labelled with H2A-GFP and transplanted into host flies to assess their tumorigenic potential^15^. The transplantation of *aurA* and *aurA mad2* brain tissues induced tumour formation in 82% (41/50) and 80% (45/56) of cases. (**f**) The transplantation of H2A-GFP-labelled *sas-4* brain tissues led to tumour formation (5.9%, 6/102) whereas the transplantation of *sas-4 mad2* brain tissues did not (0%, 0/112). Scale bar, 0.5 mm.

**Figure 2:**
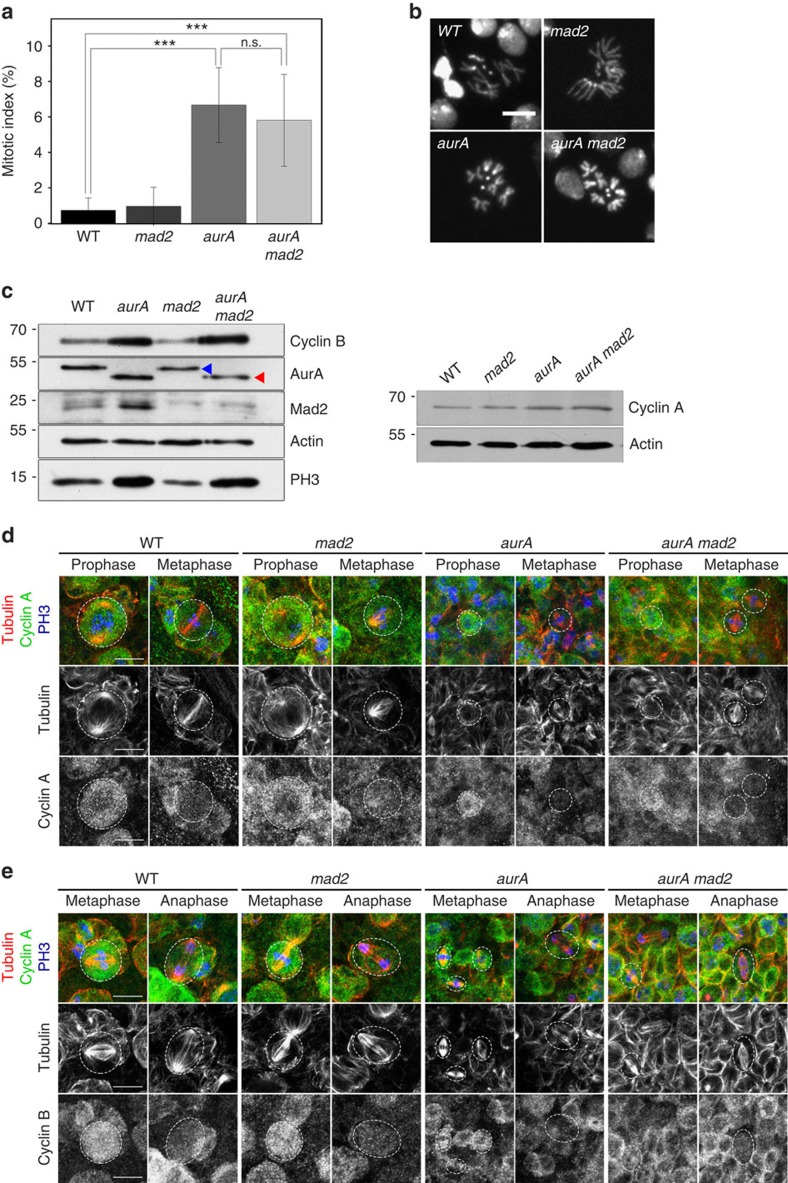
Mitotic delay in *aurA* mutants is SAC independent. (**a**) Analysis of mitotic indices (±s.d.) in WT, *mad2*, *aurA* and *aurA mad2* neural tissues. WT: 0.73±0.69% (*n*=11,332, three brains); *mad2:* 0.96±1.07% (*n*=9,507, three brains); *aurA*: 6.7±2.1% (*n*=11,334, three brains); and *aurA mad2*: 5.8±2.8% (*n*=8,648, three brains). ***P<10^−10^ (Wilcoxon test). (**b**) Chromosome morphology in WT, *mad2*, *aurA* and *aurA mad2* neural tissues. Note that in both *aurA* (bottom left) and *aurA mad2* (bottom right) mutants, the mitotic cells have highly condensed chromosomes, indicative of a delay in mitosis, unlike WT and *mad2* mutant brain cells (top). Scale bar, 10 μm. (**c**) Western blots showing the levels of cyclin B, AurA, Mad2, phospho-histone H3 (Ser10) (left) and cyclin A (right). Actin was used as a loading control. Cyclin B and phospho-histone H3 (Ser10) are high in *aurA* and *aurA mad2* neural tissue extracts, whereas cyclin A protein levels are not. The truncated AurA^K377/STOP^ corresponding to the *aurA*^*8839*^ mutation is detected as a higher mobility protein in the corresponding *aurA* and *aurA mad2* brain extracts (red triangle), whereas the endogenous Aurora A is indicated by the blue triangle. (**d**) Cyclin A degradation occurs during prometaphase in WT, *mad2*, *aurA* and *aurA mad2* mitotic cells. WT and mutant brains of the indicated genotypes were fixed and stained for cyclin A (green and lower monochrome panels) during G2 and prometaphase. Microtubules are labelled in red (and on the middle monochrome panels) and phospho-histone H3 (Ser10) is displayed in blue. (**e**) Cyclin B degradation occurs at the metaphase to anaphase transition in WT, *mad2*, *aurA* and *aurA mad2* mutant brains. Brains were fixed and stained for cyclin B (green and lower monochrome panels), tubulin (red and middle monochrome panels) and phospho-histone H3 (Ser10). Scale bar, 10 μm.

We therefore created various double-mutant lines for *aurA*^*8839*^ and *mad2*, and checked the double mutation by confirming that the Mad2 protein was absent and that there was a higher mobility form of the truncated AurA^K377/stop^ mutant protein (Fig. 2c, second panel from the top). We confirmed that the null *mad2*^*P*^ mutant (hereafter referred to as *mad2*) was viable^4^,^14^. In *mad2* larval brains, the number of NBs (±s.d.) (73.2±9.0 NBs per lobe, *n*=22) was similar to that in the wild type (75.4±10.5 NBs per lobe, *n*=14) (Fig. 1a left panels and b).

Surprisingly, the *aurA mad2* double mutant displayed marked brain overgrowth, similar to that observed for *aurA* mutants (Supplementary Fig. 5a). Consistent with the overgrowth phenotype, the larval brains of both *aurA* and *aurA mad2* individuals had more NBs per lobe than the WT (Fig. 1a right panels and c). We labelled larval brain tissues of single- and double-mutant brains with H2A-green fluorescent protein (GFP), dissected them and injected them into host flies to assess their ability to induce tumour formation^15^. Tumour formation rates were similar for *aurA mad2* double-mutant brains (∼80%, 45/56 injected flies, Fig. 1e) and for *aurA* single-mutant brains (82%, 41/50). By contrast, neither WT nor *mad2* mutant brains triggered tumour formation, consistent with published findings^5^. We confirmed these results with another *aurA* allelic combination (*aurA*^*8839*^*/aurA*^*17961*^), and with a *bubR1* allele causing a defective SAC (hereafter named *bubR1-KEN* (ref. 16)). Like *aurA mad2* brain tissues, *bubR1-KEN aurA*^*8839*^*/aurA*^*17961*^ brains were bigger than WT, contained large numbers of NBs and had high mitotic indices (Supplementary Fig. 1).

We investigated whether the ability of *aurA* neural tissues to develop tumours even in the absence of a SAC was a general feature of mutants generating supernumerary NBs, by studying the consequences of inactivating the SAC in *sas-4* mutants. The spindles of *sas-4* mutants lack centrioles and astral microtubules, and they display alignment defects similar to those seen in *aurA* mutants^4^,^10^,^17^ (Fig. 1d). In *sas-4* NBs, spindles assemble from microtubules that have randomly nucleated around chromatin, and the duration of mitosis is prolonged by 30–40%, presumably due to the action of the SAC^4^. Thus, while *sas-4* mutants display very low levels of aneuploidy, their spindle orientation is defective and their brains can generate tumours on transplantation^5^.

We confirmed that the *sas-4*^*s2214*^ mutant (hereafter referred to as *sas-4*) was viable^4^,^14^. The brains of *sas-4* mutants contained about 70% more NBs than WT brains (126.2±16.2 per lobe, *n*=18, *P*=7.9 × 10^−8^, Wilcoxon test), consistent with their ability to develop into tumours when injected into host flies^5^ (Fig. 1f). However, double-mutant *sas-4 mad2* individuals had much smaller brains, with far fewer NBs (42.0±14.5 NBs per lobe, *n*=14; Fig. 1a,b), and their brain tissues did not form tumours after injection into host flies (Fig. 1f). In addition, dividing NBs were only very rarely observed in the double mutants, and almost all these cells had abnormal spindle structures (Fig. 1d). Similarly, a *bubR1-KEN sas-4* double mutant was found to have impaired tissue growth and smaller than normal numbers of NBs (Supplementary Fig. 2a,b).

### High mitotic indices and normal ploidy in *aurA mad2* brains

Inactivation of the SAC in *sas-4* mutants severely impaired brain development and triggered spindle assembly failure, whereas such inactivation had little effect in *aurA* mutants. The growth defects in the *sas-4 mad2* mutant were associated with the presence of numerous aneuploid (42.6%) and polyploid (35.5%) cells. By contrast, the rates of aneuploidy and polyploidy were only slightly increased by SAC ablation in *aurA* mutants (Supplementary Fig. 3). These opposite results for *sas-4* and *aurA* mutant NBs suggest that a specific mechanism operates in *aurA* mutant NBs (but not in *sas-4* mutant NBs), allowing correct chromosome segregation to occur even if the SAC is not functional.

The larval brains of *aurA* mutant individuals have a high mitotic index (6.7%, versus about 1% in the WT)^11^ (Fig. 2a). This high mitotic index is presumably due to the intervention of the SAC, because *aurA* mutant spindle assembly is defective. Surprisingly, however, *aurA mad2* double mutants had a mitotic index of 5.8±2.8%, very similar to that of *aurA* brains (Fig. 2a). As described previously, *aurA* mitotic NBs divide symmetrically to generate smaller NBs^7^. In addition, the mitotic cells of both *aurA* and *aurA mad2* individuals presented highly condensed chromosomes with preserved centromeric cohesion (Fig. 2b), a feature characteristic of a prolonged M phase^18^. Cyclin A protein levels were normal (Fig. 2c, right) and this protein was degraded normally, during prometaphase, in all genotypes examined (Fig. 2d). However, phosphorylated histone (phospho-histone) H3 (Ser10) and cyclin B protein levels were higher in *aurA mad2* and *aurA* brain extracts than in WT or *mad2* extracts (Fig. 2c, left). Analyses of fixed specimens from *aurA* and *aurA mad2* mutants confirmed the high frequency of mitotic cells labelled for phospho-histone H3 (Ser10) and cyclin B. However, cyclin B was ultimately degraded in all anaphase cells examined, for both genotypes (*n*>20; Fig. 2e). Finally, we observed no lagging chromosomes during anaphase and telophase in fixed preparations of tissues from *aurA* and *aurA mad2* mutants (*n*>40). These findings suggest that mitotic exit and cyclin B degradation were delayed in a SAC-independent manner in *aurA mad2* mutant NBs, and that this delay was sufficient to allow correct attachment of the chromosomes to the spindle before the onset of anaphase.

### A delay between SAC satisfaction and anaphase in *aurA* NBs

**Figure 3:**
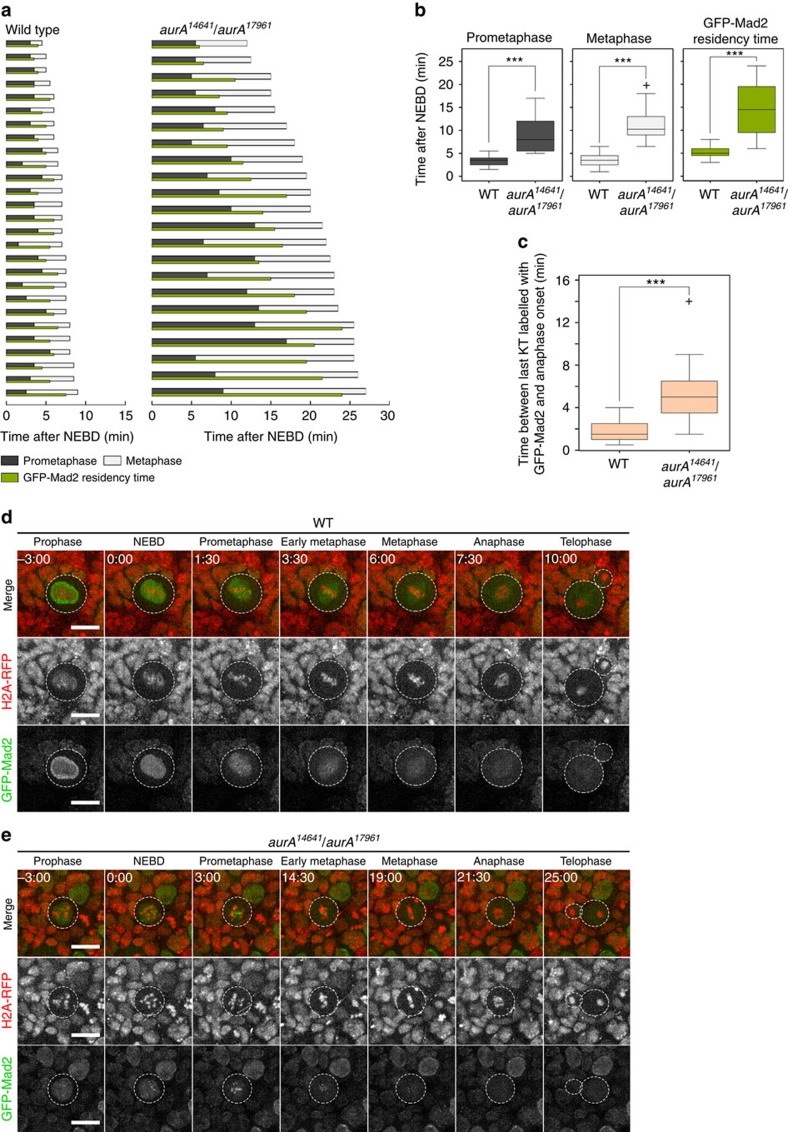
Metaphase to anaphase transition is delayed in *aurA* hypomorphic mutant NBs. (**a**) Mitotic timing and spindle assembly checkpoint analysis in 26 WT (left) and 22 *aurA*^*14641*^*/aurA*^*17961*^NBs (right). The grey and white bars indicate the time spent by each cell in prometaphase and metaphase, respectively. The green bar indicates the time for which GFP-Mad2 was present at the kinetochores. (**b**) Box plots showing prometaphase duration (left, WT: 3.27±0.93 min; *aurA*^*14641*^*/aurA*^*17961*^: 8.81±3.42 min; *P*=1.2 × 10^−11^, Wilcoxon test), metaphase duration (middle, WT: 3.7±1.3 min; *aurA*^*14641*^*/aurA*^*17961*^: 11.6±3.8 min; *P*=1.0 × 10^−11^, Wilcoxon test) and Mad2 occupancy time at the kinetochore (right, WT: 5.2±1.1 min; *aurA*^*14641*^*/aurA*^*17961*^: 14.6±5.4 min; *P*=3.0x10^−13^, Wilcoxon test) in WT (*n*=54) and *aurA*^*14641*^*/aurA*^*17961*^ (*n*=22) NBs. The above values are the means±s.d. Box plot: boxes show the upper and lower quartile. *P* values are determined with a Wilcoxon test. (**c**) Box plots showing the time interval between SAC satisfaction and mitotic entry. In WT cells, the time between SAC satisfaction and anaphase onset was 1.76±0.94 min (*n*=54). By contrast, this interval was much longer, and significantly so, in the *aurA* mutant, at 5.75±1.88 min (*n*=22). ***P=1.9 × 10^−10^, Wilcoxon test. Box plot: boxes show the upper and lower quartile. *P* values are determined with a Wilcoxon test. (**d**,**e**) RFP-histone H2A was used to monitor chromosome dynamics (red in the top panels and in the monochrome middle panels), and GFP-Mad2 (green and bottom lower monochrome panels) was used to monitor chromosome attachment and SAC satisfaction. Scale bars, 10 μm. Time (min:s) is indicated at the top left of each image. NEBD began at 00:00. (**d**) Analysis of chromosome and GFP-Mad2 dynamics in WT NBs. (**e**) Analysis of chromosome and GFP-Mad2 dynamics in *aurA*^*14641*^*/aurA*^*17961*^ NBs.

We characterized this unexpected phenotype further by assessing SAC activation by following GFP-Mad2 recruitment and the timing of cyclin B degradation in live *aurA* mutant NBs. The mitotic delay was very long (∼60 min) for the *aurA*^*8839*^ allele. We therefore also used a combination of less severe alleles (*aurA*^*14641*^*/aurA*^*17961*^) (ref. 7), making it possible to minimize the bleaching of red fluorescent protein (RFP)-Histone H2A and GFP-tagged proteins during the time course of the experiments (Fig. 3a,b). In WT NBs, GFP-Mad2 labelled the nuclear envelope and nucleoplasm before mitosis, and weak fluorescence was observed at the kinetochores during prometaphase (Fig. 3d and Supplementary Movie 1), consistent with previous findings^19^,^20^. In all WT cells examined, this weak GFP-Mad2 signal at the kinetochores completely disappeared 1.76±0.85 min (*n*=55) before anaphase onset (Fig. 3c). In *aurA*^*14641*^*/aurA*^*17961*^ mutant NBs, GFP-Mad2 strongly labelled the kinetochores, consistent with defective kinetochore–microtubule attachment and strong SAC activation (Fig. 3a,b,e and Supplementary Movie 2). This strong GFP-Mad2 kinetochore signal decreased slowly in intensity as the chromosomes congressed to the metaphase plate, indicating a delay in the establishment of correct kinetochore–microtubule attachments (Fig. 3e, top and Supplementary Movie 2). However, the time lag between the disappearance of GFP-Mad2 from the kinetophores and the onset of anaphase was significantly longer (5.75±1.88 min, *n*=22) in all dividing *aurA*^*14641*^*/aurA*^*17961*^ cells than in WT cells (Fig. 3c).

### Cyclin B degradation is impaired in *aurA* mutant NBs

**Figure 4:**
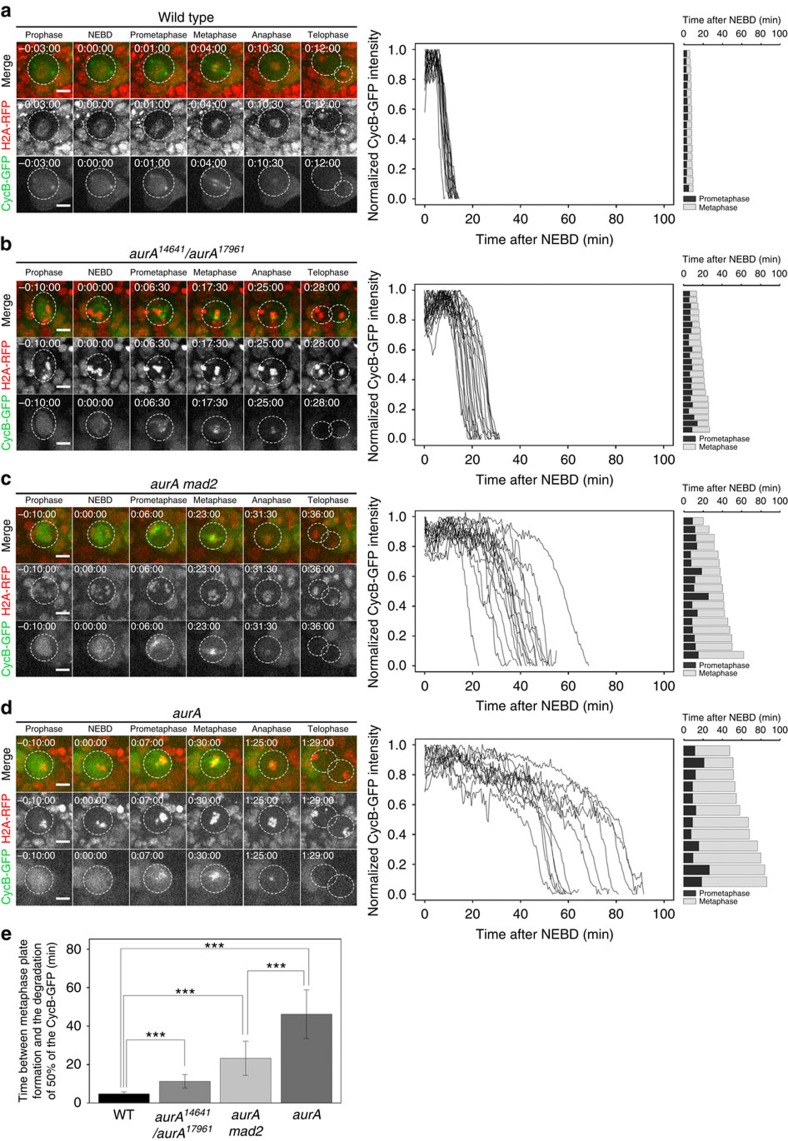
Cyclin B degradation is delayed in a SAC-independent manner in *aurA* NBs. (**a**–**d**) (Far left column panels) RFP-histone H2A was used to monitor chromosome dynamics (red in the top panels and in the middle monochrome panels), and cyclin B-GFP was used to determine the amounts of cyclin B protein in individual cells (green and lower monochrome panels). The panels of the middle column show the cyclin B-GFP degradation profiles of the corresponding genotypes. The timing of mitosis (far right column panels) is indicated for each individual NB. The black and grey bars indicate the durations of prometaphase and metaphase, respectively. Time (h:min:s) is indicated at the top left of each image. NEBD began at 00:00:00. Scale bars, 5 μm. (**a**) Timing of mitosis and cyclin B-GFP degradation in WT NBs (*n*=18). (**b**) Timing of mitosis and cyclin B-GFP degradation in *aurA*^*14641*^*/aurA*^*17961*^ NBs (*n*=23). (**c**) Timing of mitosis and cyclin B-GFP degradation in *aurA mad2* double-mutant NBs (*n*=17). (**d**) Timing of mitosis and cyclin B-GFP degradation in *aurA* null mutant NBs (*n*=12). (**e**) Graph showing the mean time (±s.d.) to 50% cyclin B-GFP degradation for WT, *mad2*, *aurA mad2* and *aurA* NBs. WT: 4.8±1.1 min (*n*=18); *AurA*^*14641*^*/aurA*^*17961*^: 10.3±3.4 min (*n*=23); *aurA*: 46.2±12.6 (*n*=12); and *aurA mad2*: 23.3±8.9 min (*n*=17). ***P<7 × 10^−5^ (Wilcoxon test).

Anaphase onset is directly connected to anaphase promoting complex/cyclosome (APC/C) activation and the degradation of mitotic targets, including cyclin B and securin, therefore we monitored cyclin B-GFP degradation in *aurA* and *aurA mad2* mutants. Consistent with previous findings, cyclin B-GFP accumulated at the centrosome and in the nucleus of WT NBs before entry into mitosis. Following NEBD, cyclin B-GFP was visible throughout the cell, but the fluorescence was particularly intense in the kinetochore region and mitotic spindle^19^,^21^ (Fig. 4a and Supplementary Movie 3). The cyclin B-GFP signal began to disappear abruptly, shortly after the last chromosome reached the metaphase plate, and half the cyclin B-GFP present was degraded 4.75±1.04 min (*n*=18) after metaphase plate formation (Fig. 4e). The cells of the *aurA*^*14641*^*/aurA*^*17961*^mutant took 11.3±3.5 min (*n*=23) to degrade half their cyclin B (Fig. 4b,e and Supplementary Movie 4), whereas *aurA* null mitotic cells took 46.2±12.6 min (*n*=12; Fig. 4d,e and Supplementary Movie 6). The introduction of the *mad2* mutation in the *aurA* null mutant shortened the time required for half the cyclin B to be degraded to 23.2±8.9 min (*n*=17) but did not restore the WT timing of mitosis (Fig. 4c,e and Supplementary Movie 5). Consistent with observations in fixed anaphase specimens, live imaging of *aurA* and *aurA mad2* mutant cells showed no lagging/delayed chromatids during anaphase that might have resulted from incorrect attachment^16^,^22^. This suggests that the machinery responsible for correcting inappropriate attachments between kinetochores and spindle microtubules is fully functional. As cells with Aurora A defects display delayed mitosis, we investigated whether AurA overexpression could accelerate mitosis, as in some cancer cell lines^23^. However, we found that mitotic timing was normal under normal conditions in NBs overexpressing Aurora A kinase, with no acceleration (Supplementary Fig. 4). Furthermore, these NBs remained arrested in M phase in the presence of microtubule depolymerizing drugs (unpublished observations).

### *aurA* mutation restores the euploidy of *sas-4 mad2* brains

Our results suggest that *aurA* mutant NBs display defective cyclin B degradation, resulting in a SAC-independent delay of mitosis. If this is indeed the case, then introducing the *aurA* mutation in a *sas-4 mad2* background should (to some extent) restore mitotic timing and euploidy (by lengthening the M phase and correcting the defective kinetochore/microtubule attachments). Consistent with this hypothesis, we found that the mitotic index increased from 0.6% in the *sas-4 mad2* mutant to 4.7% in the *aurA sas-4 mad2* triple mutant (Supplementary Fig. 5d). In addition, only 21.9% of the cells had the correct number of chromosomes in *sas-4 mad2* double mutants, whereas most of the mitotic *aurA sas-4 mad2* cells (63.9%) displayed normal ploidy (Supplementary Fig. 3c). Furthermore, brain overgrowth and NB amplification were also detected in this triple-mutant background (Supplementary Fig. 5a–c).

## Discussion

This report suggests that Aurora A may be required for the correct APC/C-dependent degradation of cyclin B. The SAC is transiently activated in *aurA* mutants (as shown by the strong recruitment of GFP-Mad2 to kinetochores), but this is not sufficient to account for the observed delay in mitosis. Our genetic and live imaging studies indicate that the delay in *aurA* mutant cells has two components, one dependent on the SAC and the other independent of the SAC. In *Drosophila*, mutations affecting APC/C subunits lead to poor CNS development and an accumulation of the mitotic cyclins A and B^24^,^25^. Cyclin A levels are normal and this protein is degraded normally in *aurA* mutant tissues. It is therefore unlikely that overall APC/C activity is affected. Instead, we favour the hypothesis that some, but not all, APC/C substrates (the most important of which being cyclin B) are more stable in *aurA* and are not efficiently ubiquitylated. The underlying mechanism remains unclear, but one recent report revealed that *Drosophila* cyclin B is itself a substrate of Aurora B kinase during abscission, raising the possibility that cyclin B could be a target of Aurora A kinase. However, it seems unlikely that such phosphorylation events could control the correct timing of mitosis, as mitotic timing was found to be normal in dividing germline cells expressing non-phosphorylatable version of cyclin B^26^. Interestingly, a similar pattern of SAC-independent mitotic arrest has also been described for polo-depleted S2 cells^27^. In cultured cells, human AurA phosphorylates the activation loop of Plk1, triggering entry into mitosis^28^. In addition, as the CNS of *polo* mutants also displays NB amplification and tumour formation^5^, it is tempting to speculate that an AurA/Polo kinase cascade could control not only the timely entry into mitosis but also other mitotic events, including cyclin B degradation. APC/C subunits have many sites for *in vivo* phosphorylation by various kinases, including Plk1 and Aurora A^29^,^30^. A complex network of phosphorylation/dephosphorylation events is probably required to promote the efficient degradation of APC/C substrates at the onset of anaphase, but Aurora A is undoubtedly a key player in this process.

In flies and human cell lines, Mad2 depletion accelerates the passage through mitosis^14^,^31^. Consistent with a role for Aurora A in the regulation of cyclin B degradation, it has been suggested that the overproduction of Aurora A in one human cancer cell line overrides the SAC, leading to premature mitotic exit^23^. However, we found that NBs overexpressing Aurora A kinase had normal mitotic timing. Thus, by contrast to findings for human cancer lines, our results favour a model in which a pool of Aurora A functions normally downstream from or in parallel to the SAC, promoting the correctly timed and efficient degradation of cyclin B in fly neural stem cells.

This study reveals, for the first time, that compromised Aurora A activity can prevent chromosome segregation defects and subsequent massive aneuploidy in a SAC-independent manner. This effect seems to be specific to *aurA* mutations, as *sas-4* mutant NBs (which have similar spindle assembly and orientation defects), display severe impairment following SAC inactivation. For brain homeostasis, defective Aurora A activity therefore has disastrous consequences. First, it leads to the generation of many NB-like cells, due to a loss of cell polarity and to incorrect spindle orientation, leading to tumour formation. Second, the prolonged SAC-independent delay in mitosis enables *aurA* cells to avoid the potentially deleterious chromosome segregation defects observed for other mitotic mutants in the absence of the SAC.

Aurora A kinase is a target of several anticancer compounds currently undergoing preclinical trials. However, not all types of cancer seem to respond to Aurora A inhibitors^32^,^33^. We describe here an unexpected regulatory mechanism according to which Aurora A inhibition in fly neural stem cells leads to a delay in the progression of mitosis that is sufficiently long to allow the chromosomes to segregate correctly. It will therefore be of great interest to determine whether this mechanism is conserved in humans and whether it affects the relevance of Aurora A-based anticancer treatments in some types of tumours.

## Methods

### Live microscopy and immunofluorescence analysis

Third-instar larval brains were dissected in Schneider media and NBs were processed for live imaging or immunofluorescence analyses, as previously described^12^. Cyclin B-GFP degradation kinetics were analysed in individual NBs, with a published method for human cultured cells and fly NBs^14^,^34^. Images were acquired with a spinning-disk system mounted on an inverted microscope (Elipse Ti; Nikon) equipped with a × 60 1.4 NA (numerical aperture) objective at 25 °C. Z series were acquired every 30 s, with a sCMOS ORCA Flash 4.0 (Hamamatsu) controlled with MetaMorph acquisition software version X. Images were processed for quantification with ImageJ software and are presented as maximum-intensity projections. GFP fluorescence was quantified for the whole cell. The signal was adjusted for background and for bleaching relative to the signal of a neighbouring non-mitotic cell (for which the signal was assumed to be constant). In the graphs, signal levels for the whole cell are displayed as normalized signals relative to the maximal intensity measured for the cell.

The brain squash analysis protocol for counting mitotic cells has been described elsewhere^35^. Briefly, brains were dissected in PBS and incubated for 8 min in a 0.5% sodium citrate solution on a glass slide. The brains were then incubated for 30 s in 45% acetic acid and 2 min in 60% acetic acid, squashed with a clean, silicon-coated coverslip and frozen in liquid nitrogen. Once the coverslip had been removed, the preparations were briefly washed in PBS and mounted in Prolongold +1 μg ml^−1^ 4,6-diamidino-2-phenylindole (Life Technologies). They were visualized with a Leica DMRXA2 microscope (objective × 63, NA 1.32) and images were acquired with a CooSnap ES camera and Metamorph software (Roper Scientific). The images were processed with ImageJ. The mitotic index was calculated by determining the number of mitotic figures per optic field, every four fields. At least three brains were examined for quantification.

### Antibodies and western blotting

The monoclonal YL1/2 rat anti-detyrosinated tubulin antibody (1:200) and the mouse monoclonal and rabbit polyclonal anti-phosphorylated histone H3 (Ser10) antibodies (1:500) were obtained from Millipore. The mouse monoclonal anti-cyclin A, anti-prospero and anti-cyclin B antibodies were purchased from the Developmental Studies Hybridoma Bank (1:10). The mouse monoclonal anti-FLAG (1:10,000) antibody was obtained from Stratagene. The rabbit anti-cyclin B antibody (1:10,000) has been described before^36^ and was kindly provided by David Glover (University of Cambridge, UK). Polyclonal rabbit anti-actin (1:4,000) antibodies were obtained from Santa Cruz, and the rat anti-deadpan antibody (1 μg ml^−1^) was obtained from Abcam. The affinity-purified rabbit anti-Mad2 polyclonal antibody (1:500) was provided by David Sharp (Albert Einstein College of Medicine, New York). Goat secondary peroxidase-conjugated antibodies (1:5,000) were obtained from Jackson ImmunoResearch Laboratories and donkey Alexa Fluor-conjugated secondary antibodies (1:1,000) were obtained from Life Technologies. For western blotting, ECL reagent was purchased from Thermo Fisher Scientific. Some of the western blots were cropped and the original scans of the blots are provided in Supplementary Fig. 6.

### Statistical analysis

Differences between data sets were assessed with a non-parametric test (Mann–Whitney–Wilcoxon), with values of *P*<0.01 considered significant.

### Fly strains

Flies were maintained under standard conditions at 25 °C. Transgenic flies with the following genotypes were used for live imaging: H2A-RFP (Bloomington Stock Center), *sas-4*^*s2214*^ (ref. 4), cyclin B-GFP^21^, *mad2*^*P*^ (ref. 14), GFP-Mad2 (ref. 19) and H2A-GFP^37^ have been described elsewhere.

*The aurA*^*8839*^ allele (hereafter referred to as *aurA*) introduces a stop codon at position 377 in the open reading frame of the kinase^7^. The resulting mutant kinase has no detectable activity *in vitro*, like the K377R (dead) mutant(7,38). The *aurA*^*14641*^/*aurA*^*17961*^ allelic combination has been described before and the corresponding fly stocks were provided by Chris Doe (University of Oregon) and Chen Yu Lee (University of Michigan)^7^. UAS-FLAG-AurA and UAS-FLAG-AurA^K/R^ plasmids were constructed by the recombination of AurA and AurA^K/R^ donor vectors (described in ref. 38) in pPFW (*Drosophila* Genomic Resources Center) with Gateway technology (Life Technologies). Transgenic flies were obtained by P element-mediated transformation. The production of tagged proteins in the fly CNS was induced with the 69B-GAL4 strain^12^.

## Additional information

**How to cite this article:** Caous, R. *et al*. Spindle assembly checkpoint inactivation fails to suppress neuroblast tumour formation in *aurA* mutant *Drosophila*. *Nat. Commun.* 6:8879 doi: 10.1038/ncomms9879 (2015).

## Supplementary Material

Supplementary Information Supplementary Figures 1-6

Supplementary Movie 1 WT dividing neuroblast expressing GFP-Mad2 and RFP-histone H2A

Supplementary Movie 2 *aurA^14641^/aurA^17961^* dividing neuroblast expressing GFP-Mad2 and RFP-histone H2A

Supplementary Movie 3 WT dividing neuroblast expressing cyclin B-GFP and RFP-histone H2A

Supplementary Movie 4 *aurA^14641^/aurA^17961^* dividing neuroblast expressing cyclin B-GFP and RFP-histone H2A

Supplementary Movie 5 *aurA mad2* dividing neuroblast expressing cyclin B-GFP and RFPhistone H2A

Supplementary Movie 6 *aurA* dividing neuroblast expressing cyclin B-GFP and RFPhistone H2A
