# Supplementary material for: Spindle assembly checkpoint inactivation fails to suppress neuroblast tumour formation in aurA mutant Drosophila
Source: Nat Commun. 2015 Nov 16;6:8879. doi: 10.1038/ncomms9879 (PMC4660220; doi:10.1038/ncomms9879)
Supplement: Supplementary Information — Supplementary Figures 1-6 [file ncomms9879-s1.pdf]

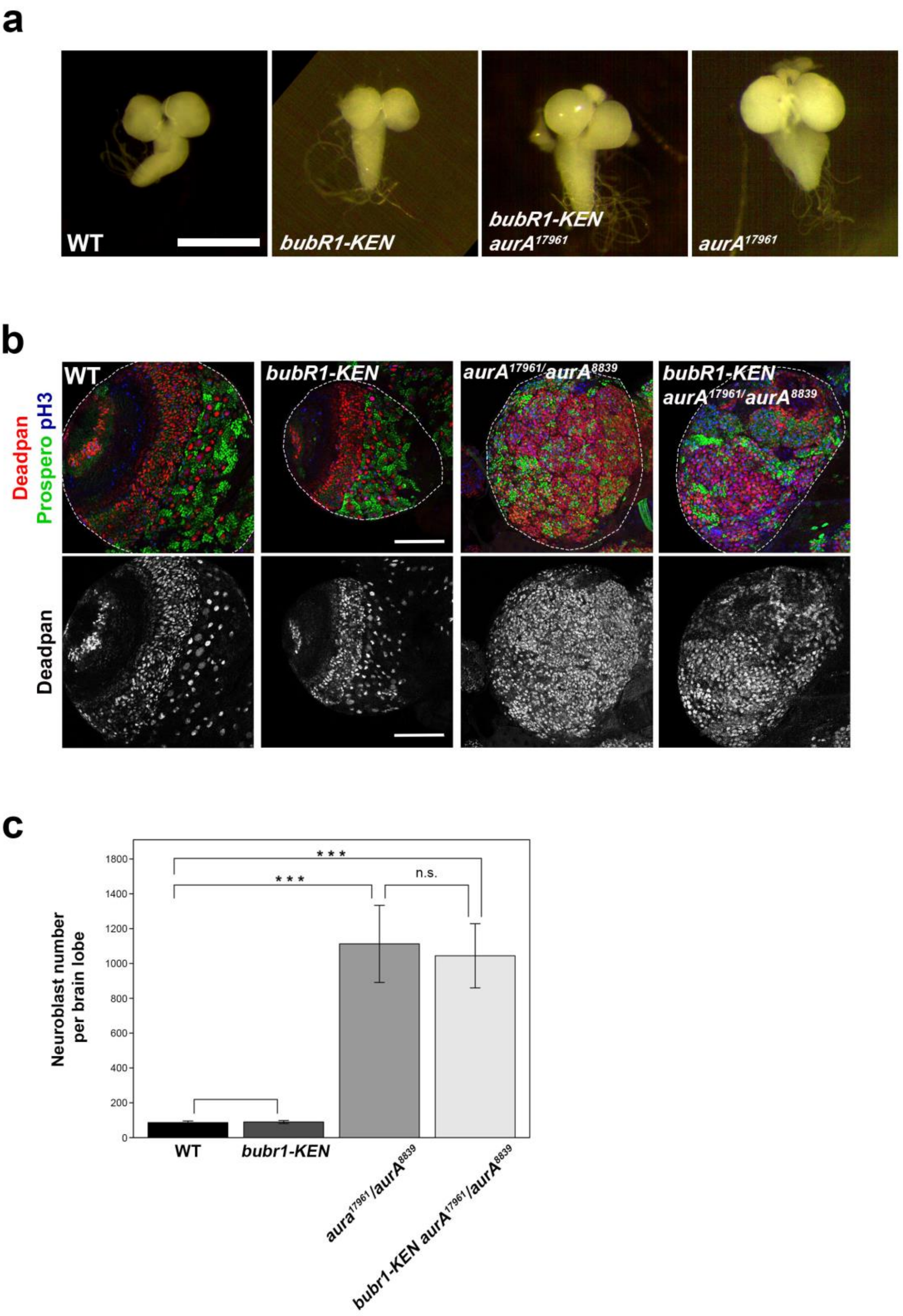

Supplementary Figure 1. *bubR1-KEN; aurA double mutants* display brain overgrowth and NB amplification

a) Brains from WT, *bubR1-KEN*, $aurA^{17961}$ and *bubR1-KEN* $aurA^{17961}$ double-mutant individuals were dissected in PBS, fixed and immediately imaged. Note the huge brain expansion in $aurA^{17961}$ and in *bubR1-KEN* $aurA^{17961}$ double mutants 144 h after larval hatching (ALH). Scale bar: 200 µm. **b**) Brains from the indicated genotypes were fixed and the numbers of pan-NBs per lobe were determined by staining of the central brain region with the anti-deadpan antibody (red and lower monochrome panels). The neuronal marker prospero is shown in green. Phospho-histone H3 (Ser10) is displayed in blue. Scale bar: 100 µm.

c) Quantification of pan-NBs numbers (± SD) in WT, *bubR1-KEN*, $aurA^{17961}/aurA^{8839}$ and *bubR1-KEN* $aurA^{17961}/aurA^{8839}$ brain lobes. WT and *bubR1-KEN* brain lobes had 86.0±9.3 NBs/lobe, (*n*=5) and 89.5±9.5 NBs/ lobe (*n*=11), respectively, whereas $aurA^{17961}/aurA^{8839}$ lobes contained 1112±221.3 NBs per lobe (*n*=7). Introduction of the *bubR1-KEN* mutation into the *aurA* defective background had no significant effect on NB number (1044.0±184.3, *n*=8, ns: *P*=0.78). ***: $P<3 \times 10^{-3}$ (Wilcoxon test).

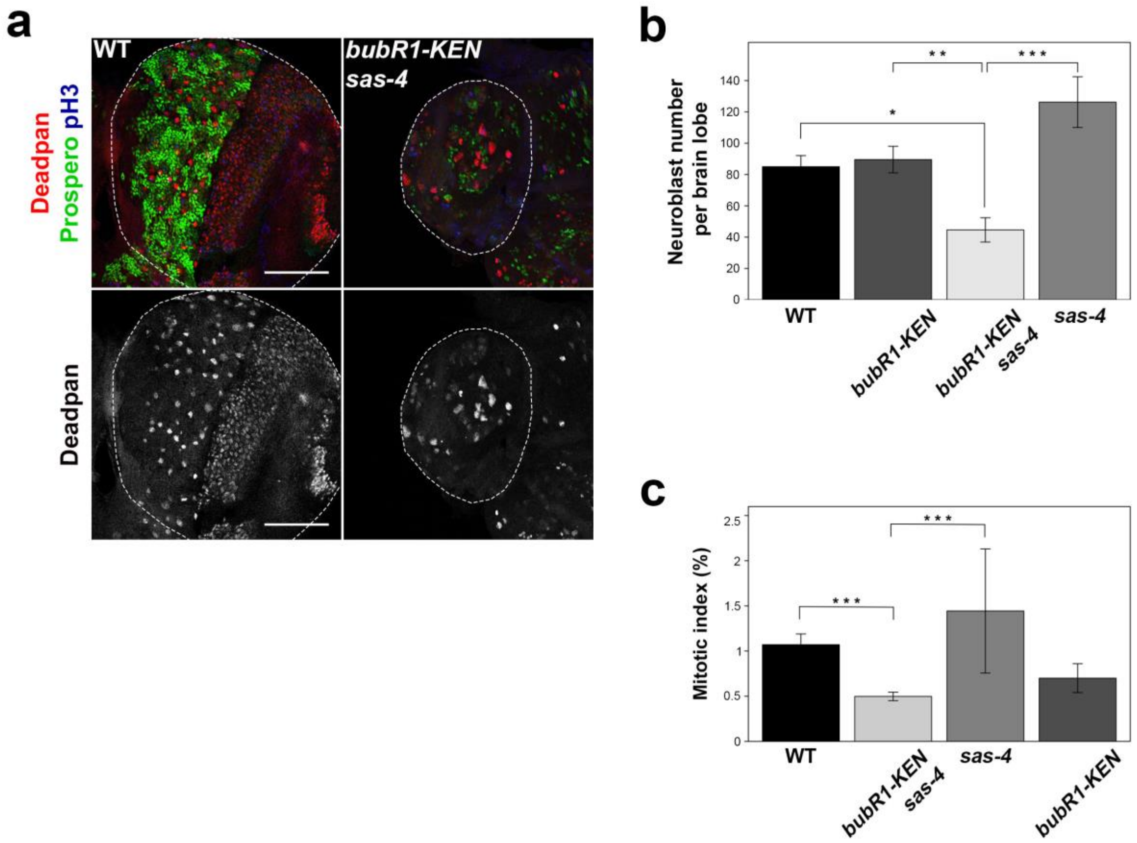

Supplementary Figure 2. *bubR1-KEN sas-4 double mutants* have smaller numbers of NBs and impaired brain growth

a) Brains from WT or *bubR1-KEN sas-4* larvae were fixed and the numbers of pan-NBs per lobe were counted by staining of the central brain region with an anti-deadpan antibody (red and lower monochrome panels). The neuronal marker prospero is shown in green. Phospho-histone H3 (Ser10) is displayed in blue. Scale bar: 100 μm.

b) Quantification of pan-NBs number (±SD) in WT, *bubR1-KEN*, *sas-4* and *bubR1-KEN sas-4* brain lobes. WT and *bubR1-KEN* brain lobes had 85.0±7.1 NBs/lobe (*n*=7) and 89.9.6±8.5 NBs/ lobe (*n*=8) respectively. *Sas-4*: 126.2±16.2 NBs/lobe (*n*=8). The introduction of the *bubR1-KEN* mutation in the *sas-4* defective background resulted in a much smaller number of NBs 44.6±7.7 NBs/ lobe (*n*=8). *: $P=1.4\times10^{-3}$, **: $P=3.3\times10^{-4}$, ***: $P<1.6\times10^{-5}$ (Wilcoxon test).

c) Analysis of mitotic indices (± SD) in WT, *sas-4, bubR1-KEN,* and *bubR1-KEN sas-4* Brains*.* WT: 1.07±0.12% (*n*=9899, 3 brains), *bubR1-KEN:* 0.70±0.16%, (*n*=5918, 3 brains), *sas-4*: 1.44±0.69%, (*n*=12417, 3 brains), and *bubR1-KEN sas-4*: 0.50±0.05% (*n*=5520, 3 brains). ***: $P<6\times10^{-6}$, (Wilcoxon test).

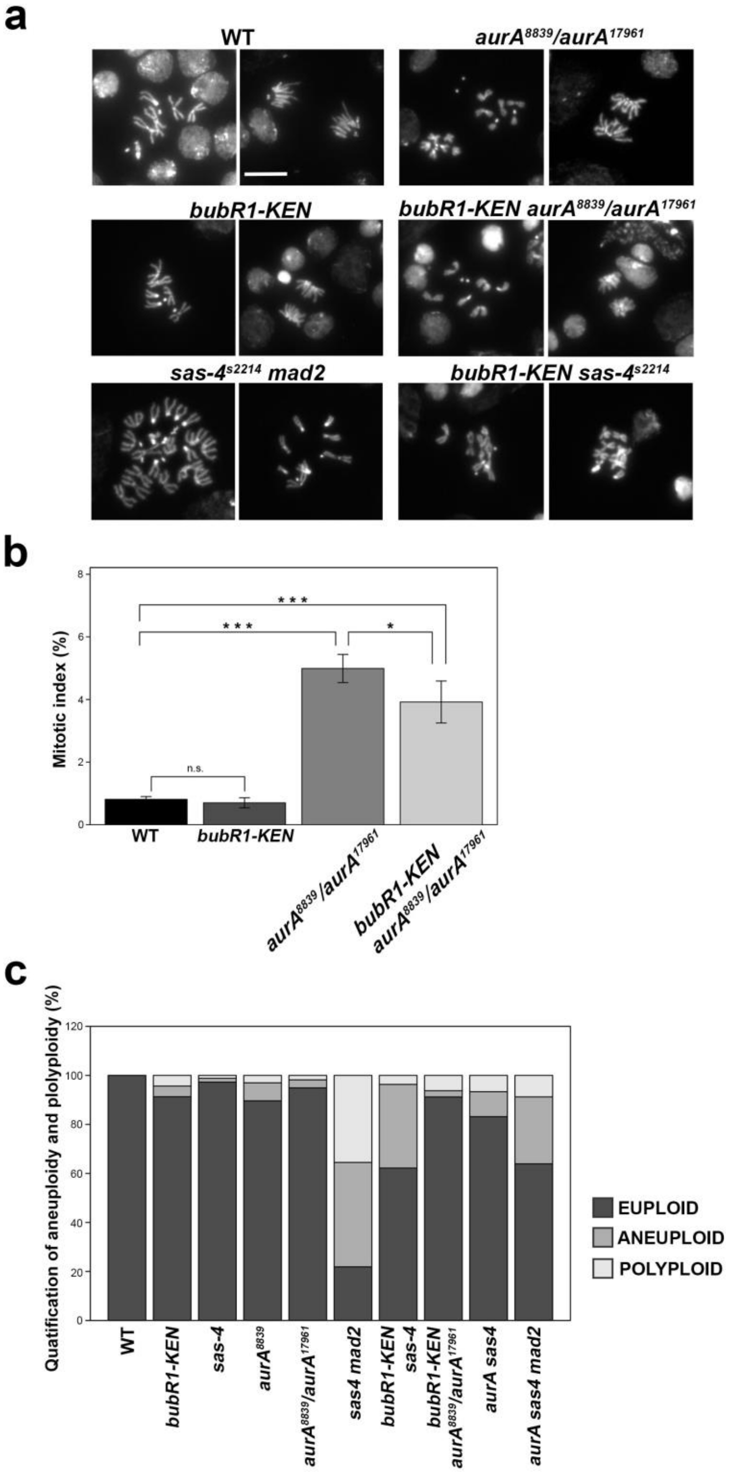

Supplementary Figure 3. Analysis of mitotic chromosome number and mitotic indices in *sas-4* and *aurA* mutant flies following disruption of the SAC

a) Selection of the most prominent mitotic figures observed in brain squashes for the genotypes indicated. The vast majority of cells are euploid in WT, *sas-4, mad2, bubR1-KEN,* and *aurA* flies. However, disrupting the SAC (*mad2* or *bubR1-KEN* mutants) in *sas-4*, but not in $aurA^{178961}/aurA^{8839}$ flies, induced severe aneuploidy and polyploidy. **b**) Quantification of mitotic indices (± SD) for the indicated genotypes.

WT: 0.81±0.09 (*n*=5326, 3 brains), *bubR1-KEN*: 0.7±0.16 (*n*=5918, 4 brains), $aurA^{8839}/aurA^{17961}$: 4.99±0.45 (*n*=7435, 4 brains), *bubR1-KEN* $aurA^{8839}/aurA^{17961}$: 3.92±0.67 (*n*=11180, 5 brains). ***: $P<2 \times 10^{-16}$, *: $P=5 \times 10^{-4}$ (Wilcoxon test). **c**) Quantification of aneuploidy and polyploidy for the indicated genotypes.

The numbers in parentheses are the percentages of euploid, aneuploid and polyploid cells, respectively; *n* is the total number of mitotic cells observed, from at least 3 different brains.

WT (100, 0, 0, *n*=150), *bubR1-KEN* (91.3, 4.4, 4.3, *n*=230), *sas-4* (97.3, 1.5, 1.2, *n*=256), $aurA^{8839}$ (89.6, 7.4, 3.0, *n*=299), $aurA^{8839}/aurA^{17961}$ (94.9, 3.3, 1.8, *n*=276), *sas4 mad2* (21.9, 42.6, 35.5, *n*=169), *bubR1-KEN sas-4* (62.2, 34.2. 3.7, *n*=82), *bubR1-KEN* $aurA^{8839}/aurA^{17961}$ (91.2, 2.6, 6.2, *n*=432), $aurA^{8839}$ *sas-4* (83.2, 10.2, 6.6, *n*=315), *aurA sas-4 mad2* (63.9, 27.3, 8.8, *n*=194).

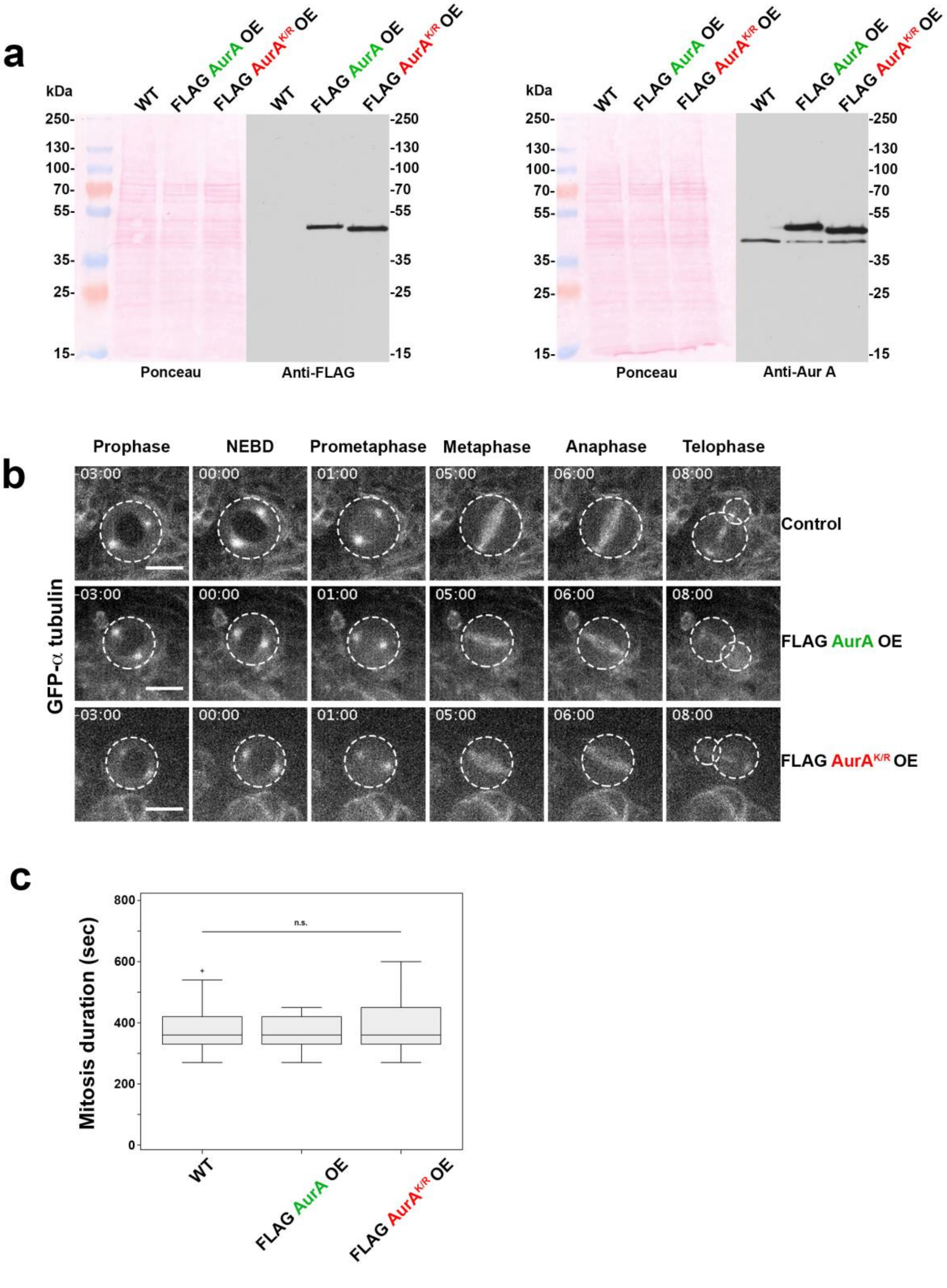

Supplementary Figure 4. AurA overexpression (OE) does not accelerate mitotic exit

a) Western blots showing the levels of endogenous and FLAG-tagged AurA variants in brain tissues. The FLAG western blot showing the overexpressed (AurA and $AurA^{K/R}$) exogenous proteins is on the left and the western blot showing both endogenous and exogenous AurA proteins is shown on the right. Ponceau S staining was used to control for loading control.

b) AurA and $AurA^{K/R}$ OE NBs display normal mitotic timing and normal spindle morphology. WT (top panels), FLAG-AurA OE (middle panels), and FLAG-$AurA^{K/R}$ OE (lower panels) NBs were imaged with GFP-α tubulin.

c) Box plot showing the duration of mitosis after overexpression of active and inactive Aurora A kinases. The duration of mitosis was not modified by the overexpression of active (FLAG-AurA OE, 371±51 s, *n*=56,) or inactive (FLAG-$AurA^{K/R}$ OE, 384±84 s, *n*=63) AurA kinases, as shown by comparison with WT NBs (382±66 s, *n*=64). Box plot: boxes show the upper and lower quartile. ns: non significant ($P>0.01$, Wilcoxon test).

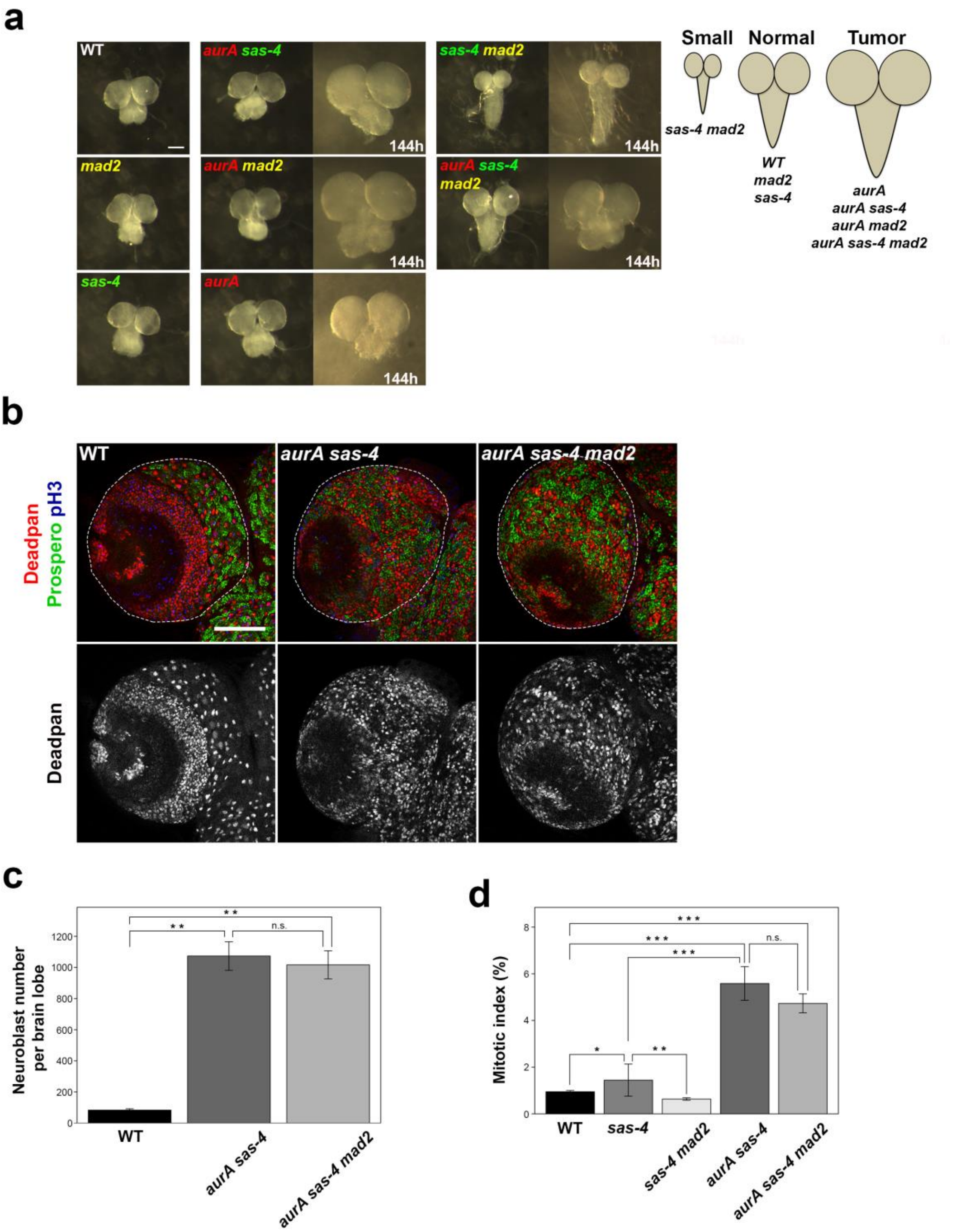

Supplementary Figure 5. *aurA* mutation restores overproliferation of the NB phenotype in the *sas-4 mad2* double mutant

a) Brains from WT, *mad2*, *sas-4 mad2*, *aur A*, *aurA sas-4, aurA mad2,* and *aurA sas-4 mad2* mutant individuals were dissected in PBS, fixed and immediately imaged. Note the impairment of brain growth following disruption of the SAC and *sas-4* (*mad2 sas-4).* The scale bar indicates 0.1 mm. The diagram on the left summarizes the various brain phenotypes associated with the genotypes examined. **b**) Brains from WT, *aurA sas-4* and *aurA sas4 mad2* triple mutants were fixed and the numbers of pan-NBs per lobe were determined in the central brain region with an anti-deadpan antibody (red and lower monochrome panels). The neuronal marker prospero is shown in green. Phospho-histone H3 (Ser10) is displayed in blue. Scale bar: 100 µm. **c**) Quantification of pan-NBs, in terms of number, in the various genetic backgrounds analyzed. WT: 83.4±8.5 NBs/lobe (n=12), *aurA sas-4*: 1073.5±91.7 NBs/lobe (n=8), *aurA sas-4 mad2:* 1016.7±90.2 NBs/lobe (n=8).**: $P<4 \times 10^{-4}$ (Wilcoxon test). **d**) Mitotic indices in WT, *sas-4, aurA sas-4, sas-4 mad2* and *aurA sas-4 mad2* neural tissues*.* WT: 0.95±0.05%, (n=5626 cells, 3 brains), *sas-4:* 1.45±0.7% (n=12417 cells, 3 brains), *sas-4 mad2*: 0.63±0.05%, (n=4962 cells, 3 brains), *aurA sas-4*: 5.6±0.7% (n=5348 cells, 3 brains) *aurA sas-4 mad2*: 4.7 ±0.4 (n=13209 cells, 3 brains). ***: $P<2 \times 10^{-16}$, ** $P<10^{-5}$, *: $P<0.03$ (Wilcoxon test).

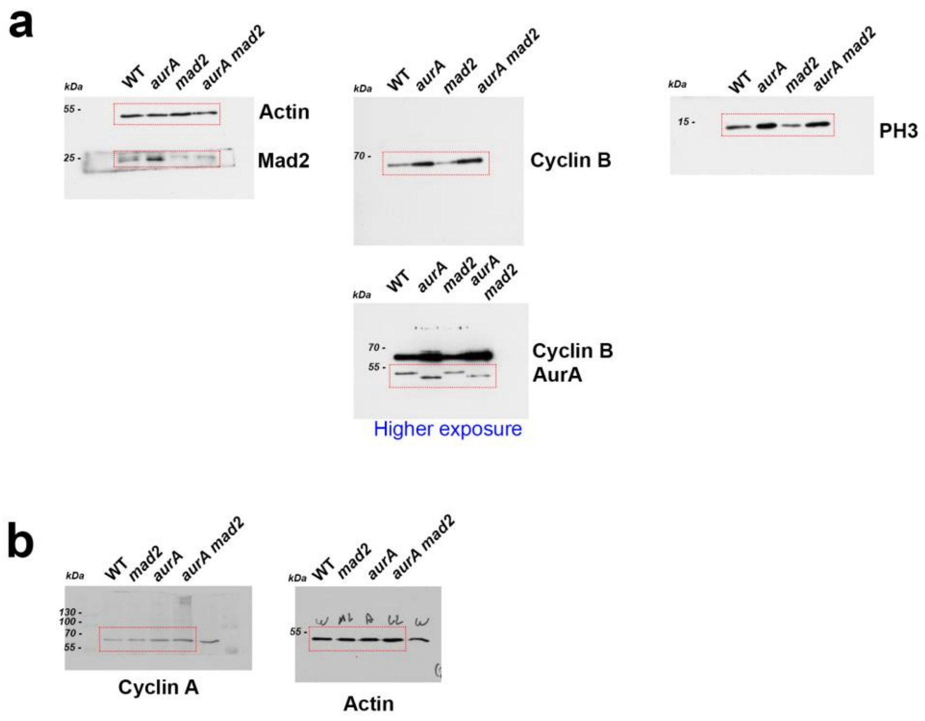

Supplementary Figure 6. Whole western blots related to Figure 2c.
Note that all the antibodies used for the western blots of this study correspond to well-characterised proteins. Therefore, one nitrocellulose membrane was often cutted (thanks to molecular weight markers) to detect 2 antigens.
a) Full western blots corresponding to the left panel of Figure 2c. Top left panel: the same nitrocellulose membrane was cutted to reveal actin (~40 kDa) and Mad2 (~24 kDa). Middle panel: the same nitrocellulose membrane was cutted to reveal cylin B (~60 kDa) and Aurora A (47Da). The middle bottom panel corresponds to longer revelation time. Right: full blot corresponding to phosphor histone H3 Ser10 (~15 kDa).
b) Full cyclin A (left) and actin (right) western blots corresponding to the right panel of Figure 2c.
The cropped region is indicated in red.
